# Comparison of surgical approaches for pectus excavatum in children and adolescents: a single-center retrospective study

**DOI:** 10.3389/fsurg.2026.1740978

**Published:** 2026-04-24

**Authors:** Sirui Ding, Xiaolong Chen, Tienan Feng, Xuequn Huang, Li Shen, Haifa Hong

**Affiliations:** 1 Cardiothoracic Surgery, Shanghai Children’s Hospital, School of Medicine, Shanghai Jiao Tong University, Shanghai, China; 2 Clinical Research Institute, Shanghai Jiao Tong University School of Medicine, Shanghai, China

**Keywords:** modified Nuss procedure, non-flipping bar, pectus excavatum, single-incision, surgical correction

## Abstract

**Objective:**

This study aimed to compare the clinical utility of two modified Nuss procedures in children and adolescents with pectus excavatum (PE), including single-incision modified Nuss and modified Nuss with non-flipping bar. It sought to delineate patient selection patterns, assess perioperative parameters, postoperative recovery, complication rates, and short/intermediate-term efficacy of the two approaches, thereby providing evidence-based guidance for individualized surgical selection.

**Methods:**

A single-center retrospective cohort study was conducted on pediatric and adolescent PE patients who underwent surgical correction at Shanghai Children's Hospital between January 2015 and December 2022. Patients were categorized by the intended operative strategy: single-incision modified Nuss (single working incision with optional thoracoscopy through the same incision) versus modified Nuss with a pre-shaped non-flipping bar (advanced without intraoperative 180° turnover). A total of 171 patients were included (140 single-incision; 31 non-flipping bar). Demographic data, clinical characteristics, surgical parameters, and postoperative outcomes were collected.

**Results:**

Regarding surgical parameters, the non-flipping bar group had longer median operative time, more incisions, and lower screw fixation rate. Postoperatively, the non-flipping bar group showed a higher median Haller index, but this difference had limited clinical significance. No significant differences were noted in overall complication rates, postoperative recovery indicators, or surgical efficacy. After PSM, no statistical difference in therapeutic efficacy was found between the two groups.

**Conclusion:**

Both the single-incision modified Nuss procedure and the modified Nuss procedure with non-flipping bar are effective and safe for PE in children and adolescents, with comparable therapeutic efficacy. Surgical selection should be tailored to patient age and deformity characteristics.

## 1 Introduction

Pectus excavatum (PE) is the most common congenital anterior chest wall deformity, characterized by posterior displacement of the sternum and adjacent costal cartilages, resulting in a funnel-shaped thorax. Its reported incidence is approximately 1 per 1,000 individuals with a male predominance, and severe deformity may lead to cardiopulmonary compression, reduced exercise tolerance, arrhythmias, and substantial psychosocial distress (1–3).

Surgical correction is considered when deformity is moderate to severe and/or associated with symptoms or objective cardiopulmonary impact. Severity is commonly quantified on cross-sectional imaging using the Haller index (HI; maximal internal transverse diameter divided by the minimal sternum-to-vertebra anteroposterior distance) and the pectus correction index (PCI; the proportion of chest depth loss relative to the ideal anterior chest wall), which facilitate standardized assessment and longitudinal follow-up (1, 4–7).

Over the past two decades, minimally invasive repair of pectus excavatum (MIRPE) based on the Nuss procedure has become the standard operative approach in children and adolescents due to smaller incisions, rapid recovery, and favorable outcomes (4, 8, 9). Nevertheless, the retrosternal tunnel creation and bar placement remain high-risk steps, and technical refinements aimed at improving visualization, mediastinal protection, and bar stabilization continue to evolve (9–13).

Among contemporary modifications, two practical variants are increasingly used in pediatric practice: (1) a single-incision modified Nuss approach, in which a single working incision is used for tunnel creation and bar passage with thoracoscopic visualization obtainable through the same incision; and (2) a modified Nuss approach using a pre-shaped non-flipping bar that is advanced in its final orientation without intraoperative 180° turnover (9, 14, 15). These strategies attempt to balance operative simplicity, safety, fixation burden, and cosmetic outcomes while adapting to patient age, deformity morphology, chest-wall rigidity, and the presence of adhesions (4, 10–12, 16, 17).

However, most published literature compares MIRPE with open repair or evaluates individual modifications in isolation, and head-to-head comparisons between modified variants remain limited, particularly in pediatric and adolescent cohorts (4, 9, 14, 18, 19). Therefore, we performed a single-center retrospective cohort study to compare the clinical utility of the single-incision modified Nuss procedure and the modified Nuss procedure with a non-flipping bar, focusing on patient selection patterns, perioperative parameters, postoperative recovery, complications, and short-to-intermediate-term efficacy. These data aim to inform individualized technique selection in routine practice.

Postoperative care is an integral component of MIRPE, particularly pain control and early mobilization. Contemporary protocols generally emphasize multimodal analgesia with regional techniques (including intercostal nerve block) and patient-controlled opioid strategies to enable effective respiration, ambulation, and shorter hospital stay (13, 20).

## 2 Data and methods

### 2.1 Data source

Technique selection was determined preoperatively by the attending surgical team based on patient age, chest-wall compliance/rigidity, deformity morphology and severity, and the anticipated retrosternal working space (including suspected adhesions), and was finalized through shared decision-making with guardians. In general, the single-incision approach was preferred in younger patients with more compliant chest walls and adequate retrosternal space, whereas the non-flipping bar strategy was more often selected in older patients and/or anatomically challenging deformities where avoiding intraoperative bar turnover was considered advantageous.

Perioperative implementation details preschool-aged children, surgery was generally reserved for clearly progressive deformity and/or clinically meaningful symptoms or objective cardiopulmonary compression, rather than cosmetic concerns alone (21). In the single-incision approach, thoracoscopy was routinely performed through the same skin incision by repositioning the trocar/camera as needed; in this study, “thoracoscopic assistance” specifically refers to placement of an additional thoracoscopic port beyond the working incision. During passage, the corrective bar traverses the pleural cavities and the retrosternal space; after placement, both bar ends ultimately lie within the lateral chest-wall soft tissue planes, and the stabilizer is positioned extrathoracically and secured to the chest wall (e.g., ribs/intercostal structures) to prevent rotation/displacement (22).

Thoracoscopic visualization was planned for all cases and was performed either through the working incision (single-incision approach) or via an additional thoracoscopic port. An additional thoracoscopic port (“thoracoscopic assistance”) was used when visualization through the working incision was suboptimal, in older patients, in severe deformity with limited retrosternal space, or when retrosternal adhesions were suspected. Bar stabilization was selected according to chest wall rigidity and bar stability after placement: a unilateral stabilizer was routinely used in the single-incision approach, whereas bilateral stabilizers (with optional gaskets) were typically used in the non-flipping bar approach; fixation was achieved with pericostal sutures and/or screws as needed. Postoperative analgesia followed a standardized multimodal protocol (scheduled acetaminophen and NSAIDs, plus patient-controlled intravenous opioids for 48–72 h) together with routine intercostal nerve block (ICNB); additional regional techniques (e.g., paravertebral blocks) were used selectively according to anesthesiologist preference and patient factors, and cryoablation was not routinely used during the study period.

**Figure 1:**
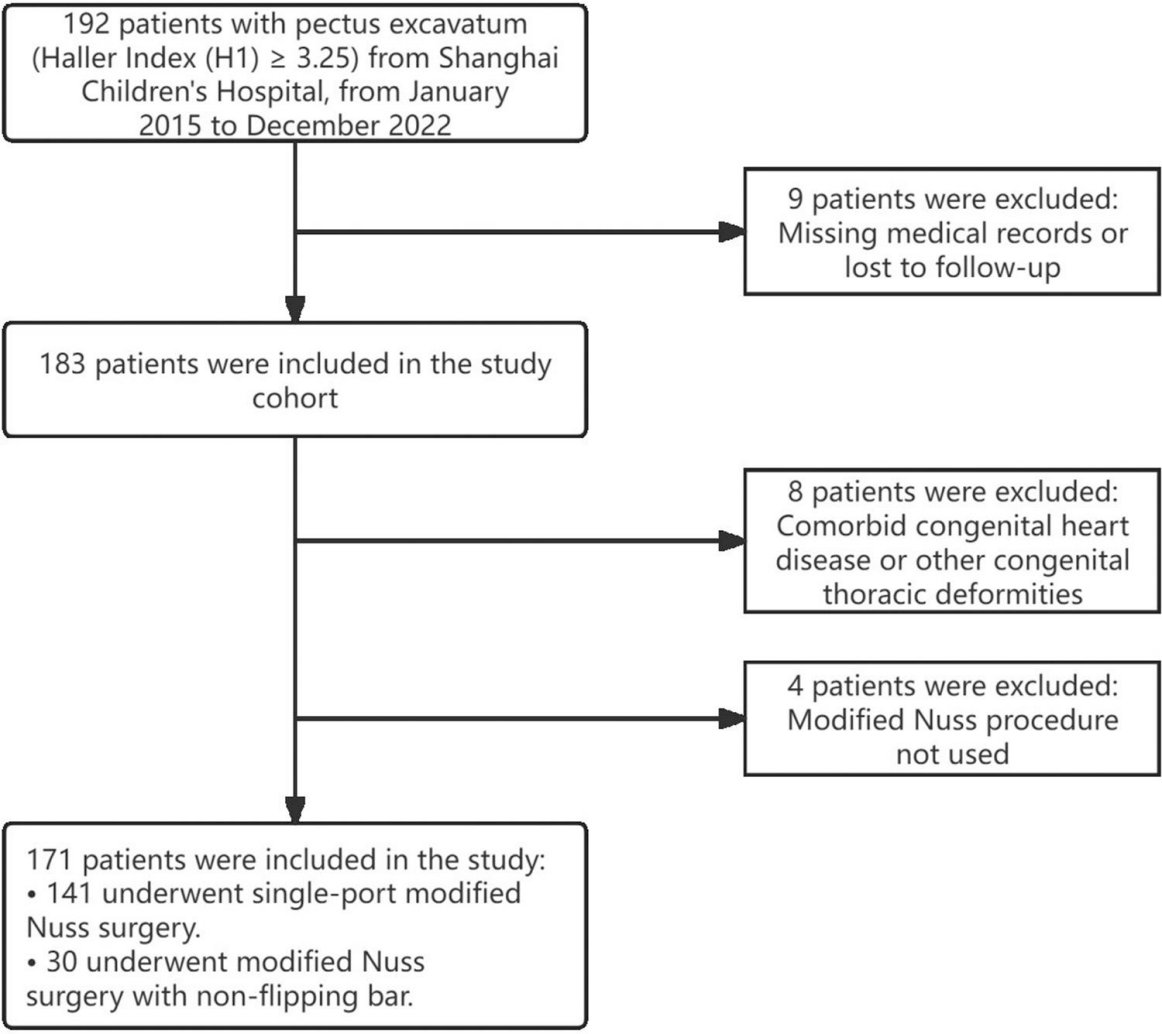
An inclusion/exclusion flow diagram.

This single-center, retrospective, observational cohort study utilized data from pediatric patients with pectus excavatum (PE) who underwent surgical correction in the Department of Thoracic Surgery at Children's Hospital of Shanghai between January 2015 and December 2022. Inclusion criteria encompassed a confirmed diagnosis of PE and eligibility for surgical intervention. Exclusion criteria were: (1) presence of severe concomitant congenital anomalies and (2) incomplete clinical documentation or loss to follow-up. Ultimately, 140 patients who received the single-incision modified Nuss procedure formed the single-incision group, while 31 patients undergoing the modified Nuss procedure with non-flipping bar comprised the non-flipping bar group (Figure 1). Surgical approach selection was based on detailed thoracic wall assessments, clinical indications, and consultations with senior attending surgeons in conjunction with parental preferences, with the aim of restoring both thoracic form and anatomical integrity. All patients were managed with standardized perioperative protocols. Preoperative evaluation routinely included chest radiography and cardiopulmonary assessment (electrocardiogram and echocardiography). Chest computed tomography (CT) was obtained selectively to quantify deformity severity (e.g., Haller index and three-dimensional reconstruction when required for operative planning) and to assess mediastinal anatomy, using institutional pediatric protocols aimed at dose minimization in accordance with the ALARA principle. Postoperatively, outpatient follow-up visits were scheduled at 1–3 months and subsequently every 3–6 months until bar removal (typically 1.5–3 years after surgery). Follow-up imaging relied primarily on chest radiographs to confirm bar position; CT was not used for routine surveillance and was reserved for predefined clinical indications (e.g., suspected bar displacement/rotation, recurrence, unexplained symptoms, or other complications) or selected pre–bar removal assessments when clinically necessary (10). To prevent interference with cardiopulmonary development arising from rapid thoracic growth, patients below 3 years of age were excluded. Individuals with complex chest wall deformities (e.g., pectus carinatum, Poland syndrome) or with traumatic sternal and rib injuries were not enrolled.

In this study, group assignment reflected the intended operative strategy at the index procedure. The “non-flipping bar” technique refers to implantation of a pre-shaped bar that is advanced through the retrosternal tunnel in its final orientation (convex side anterior) without intraoperative 180° turnover; therefore, “non-flipping” describes the implantation maneuver rather than guaranteeing absence of postoperative bar rotation/displacement. The “single-incision” technique refers to use of a single lateral working skin incision to create the presternal subcutaneous tunnel and to pass the bar; thoracoscopic visualization could be obtained through this same incision by repositioning the trocar/camera as needed. For patient safety, additional small adjunct incisions (e.g., contralateral stab incision, subxiphoid incision for digital protection/adhesiolysis, and/or access for sternal elevation) were selectively added in patients with severe deformity, limited retrosternal working space, poor chest-wall compliance, or suspected retrosternal adhesions to reduce the risk of mediastinal injury. Such cases were retained in their original group for analysis, and the frequency of additional incisions and bar-related events is reported in the Results.

Preoperative imaging routinely included posteroanterior and lateral chest radiographs, with CT reserved for quantifying deformity severity and operative planning. When CT was performed, scans were obtained without contrast and limited to the region of interest, following institutional pediatric dose-reduction practices in accordance with the ALARA principle. Postoperative surveillance was based on chest radiographs; cross-sectional imaging was obtained only when clinically indicated. When repeated cross-sectional assessment was anticipated, non-ionizing alternatives (e.g., MRI) were considered where available and appropriate.

Thoracic indices were calculated on axial cross-sectional imaging at the level of maximal sternal depression (typically near the upper xiphoid process). The Haller index (HI) was defined as the widest internal transverse diameter of the thorax divided by the minimal anteroposterior distance from the posterior surface of the sternum to the anterior surface of the vertebral body. The pectus correction index (PCI) was calculated as [(A−B)/A] × 100, where A is the maximal internal anteroposterior diameter of the thorax (from the anterior vertebral body line to the most anterior inner chest wall) and B is the minimal sternum—vertebra distance on the same slice; PCI values were recorded as a proportion (0–1) in the dataset. The cardiac compression index (CCI) was calculated as H/M, where H is the maximal transverse cardiac diameter and M is the minimal anteroposterior cardiac diameter at its narrowest point. The chest wall asymmetry index (CWAI) was calculated as L/R, where L and R represent the maximal anteroposterior diameters of the left and right hemithorax, respectively (23).

### 2.2 Data collection

All data were collected in accordance with a standardized framework encompassing four primary domains, each subjected to multilayered verification. Demographic variables (age, sex, height, and weight) were extracted from the electronic medical record (EMR) system and independently cross-checked by two reviewers for accuracy. Clinical characteristics included PE subtypes classified using three-dimensional (3D) CT reconstruction, Haller indices independently measured by two thoracic surgeons (with mean values adopted), systematic categorization of symptoms via multisystem assessments, and comprehensive histories verified through both patient interviews and previous records. Surgical parameters covered intraoperative details (e.g., operative time, blood loss) documented in real time by anesthesia monitoring systems, as well as detailed classification of postoperative complications by affected organ systems. Follow-up results incorporated quantitative analyses of thoracic morphology via standardized indices and 3D reconstruction, with symptom relief appraised based on patient-reported outcomes (PROs) and independent physician evaluations.

All acquired data underwent meticulous dual entry and cross-verification, ensuring minimized input errors. Any anomalies detected in the dataset were traced back to source documentation and verified. Core parameters, such as the Haller index, were independently and blindly re-evaluated by external third-party experts. Furthermore, prior to statistical analyses, consistency and integrity of datasets were confirmed through logical validation protocols and rigorous statistical quality checks.

### 2.3 Outcome definitions

Outcome definitions and assessment. Clinical outcomes were categorized as excellent, good, fair, or failed using prespecified criteria based on symptom change and cosmetic appearance at follow-up. “Excellent” was defined as resolution of preoperative symptoms with a normal-appearing chest contour on physical examination; “good” as resolution of preoperative symptoms with improved (but not fully normal) chest appearance; “fair” as improvement of symptoms with persistent cosmetic abnormality; and “failed” as worsening of symptoms and/or no improvement of chest appearance, or the presence of clinically significant recurrence. Cosmetic appearance was assessed by the attending surgeon at follow-up and corroborated by patient/guardian report recorded in the medical chart.

Radiological outcomes were assessed using standardized thoracic indices (e.g., Haller index and correction indices) measured on preoperative imaging and on postoperative imaging when obtained for clinical indications. “Thoracic deformity (observation)” referred to a residual or newly noted chest wall contour abnormality on clinical examination and/or chest radiograph that did not require invasive intervention during the follow-up period. “Recurrence” was defined as a reappearance or progression of sternal depression after initial correction, confirmed by clinical examination and/or imaging, and accompanied by patient/guardian concern, symptom recurrence, or the need for additional intervention (e.g., bar revision or reoperation).

### 2.4 Description of the two surgical techniques

All operations were performed under general anesthesia with the patient in the supine position and the arms abducted. Preoperative markings included the deepest point of sternal depression and the planned intercostal entry/exit level(s) for bar passage. Thoracoscopic visualization was available either through the working incision (single-incision approach) or through a separate right thoracic port (non-flipping approach); low-pressure CO2 insufflation (approximately 6–8 mmHg) could be used to improve visualization when needed (24). In our operative records, “thoracoscopic assistance” refers to the use of an additional thoracoscopic port beyond the working incision. When severe deformity or cardiac compression limited the retrosternal working space, forced sternal elevation was performed using a table-mounted retractor (e.g., Rultract) attached to a penetrating bone clamp (e.g., Lewin perforating forceps) placed on the anterior table of the sternum at the center of the defect, thereby lifting the sternum without intrathoracic penetration of the clamp tips (13). When retrosternal adhesions were suspected (e.g., redo cases) or when additional mediastinal protection was required, a small vertical subxiphoid incision was added for digital adhesiolysis and protection of the heart/pericardium during passage of the introducer (25).

### 2.5 Surgical indications and preoperative evaluation

The decision to proceed with surgical correction was made by the pediatric thoracic surgery team based on a combination of morphologic severity, clinical symptoms, and cardiopulmonary impact, with shared decision-making involving guardians. Surgical intervention was generally considered for patients with moderate-to-severe deformity (quantified on imaging, e.g., Haller index and/or correction indices), particularly in the presence of progressive sternal depression, exercise intolerance or dyspnea on exertion, chest pain, palpitations, recurrent respiratory infections, or substantial psychosocial distress attributable to the deformity (5). In younger children (=3 years), indications were applied conservatively, prioritizing symptomatic or progressive deformities and those with evidence of cardiopulmonary compromise.

Non-operative options (e.g., posture/physiotherapy and vacuum bell therapy) were discussed when appropriate; operative correction was recommended when conservative measures were not feasible or were ineffective, or when deformity severity and symptoms warranted surgery (26).

Preoperative evaluation included structured symptom assessment and review of outpatient/inpatient records, chest imaging for morphologic indices, and cardiopulmonary assessment. All patients underwent electrocardiography; transthoracic echocardiography was performed to evaluate cardiac compression/displacement and to screen for structural abnormalities. Pulmonary function testing was performed when feasible based on patient age and cooperation. Imaging acquisition and follow-up were designed to minimize radiation exposure in children in accordance with the ALARA principle (27).

### 2.6 Single-incision modified Nuss procedure

**Figure 2:**
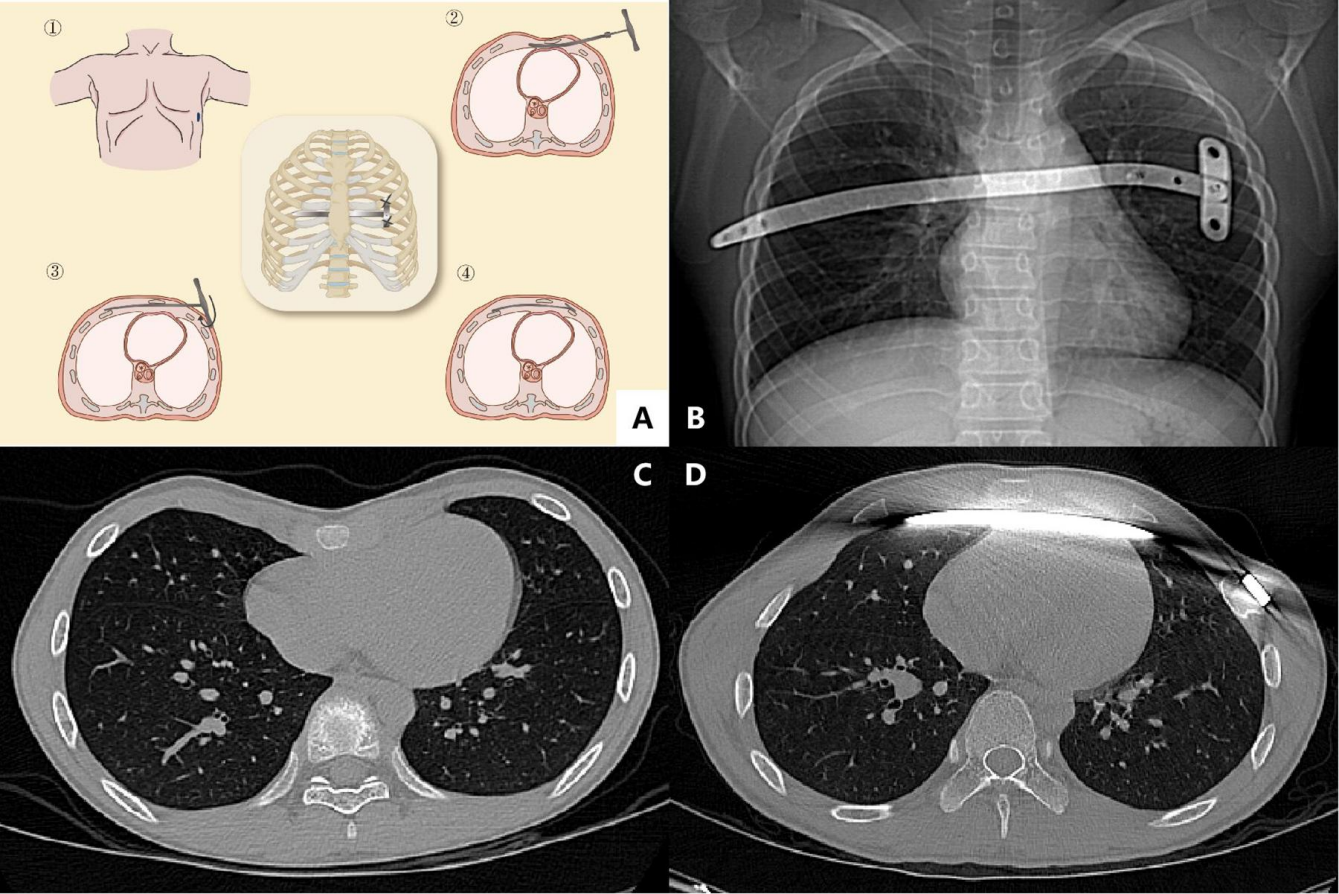
Single-incision modified Nuss procedure. **(A)** Schematic illustration of the single-incision approach showing creation of a presternal subcutaneous tunnel and retrosternal passage of the introducer/bar complex behind the sternum and anterior to the pericardium, followed by 180° bar rotation within the contralateral subcutaneous pocket. **(B)** Postoperative chest radiograph demonstrating the corrective bar with unilateral lateral stabilizer fixation. **(C)** Preoperative axial CT image at the level of maximal sternal depression. **(D)** Postoperative axial CT confirming bar position and fixation. Abbreviation: PE, pectus excavatum (15, 28).

A single 2-cm right lateral thoracic incision was made between the anterior and mid-axillary lines at the selected interspace. A generous subcutaneous tunnel was developed in a presternal, premuscular plane from the right incision toward the left lateral chest wall, creating a left-sided subcutaneous pouch wide enough to facilitate subsequent bar rotation and to minimize postoperative skin dimpling. Intrathoracic visualization was obtained using a 5-mm 45° thoracoscope introduced through the same skin incision; the trocar and camera were repositioned to two or three trajectories within the incision during retrosternal dissection and bar passage to optimize visualization. Under thoracoscopic view, the introducer/dissector was advanced through the right interspace and woven behind the sternum anterior to the pericardium, then guided into the left subcutaneous tunnel. A pectus bar approximately 2–4 cm shorter than the measured template was selected, attached to the introducer, and pulled through the retrosternal tunnel. After confirming position, the bar was rotated 180° within the left subcutaneous tunnel to elevate the sternum. The bar was secured on the right side with a unilateral stabilizer and reinforced with pericostal fixation at multiple points as needed. Before closure, the lungs were reinflated and residual pleural air was evacuated by suction and/or manual expression (28–32) (Figure 2).

### 2.7 Modified Nuss procedure with non-flipping bar

**Figure 3:**
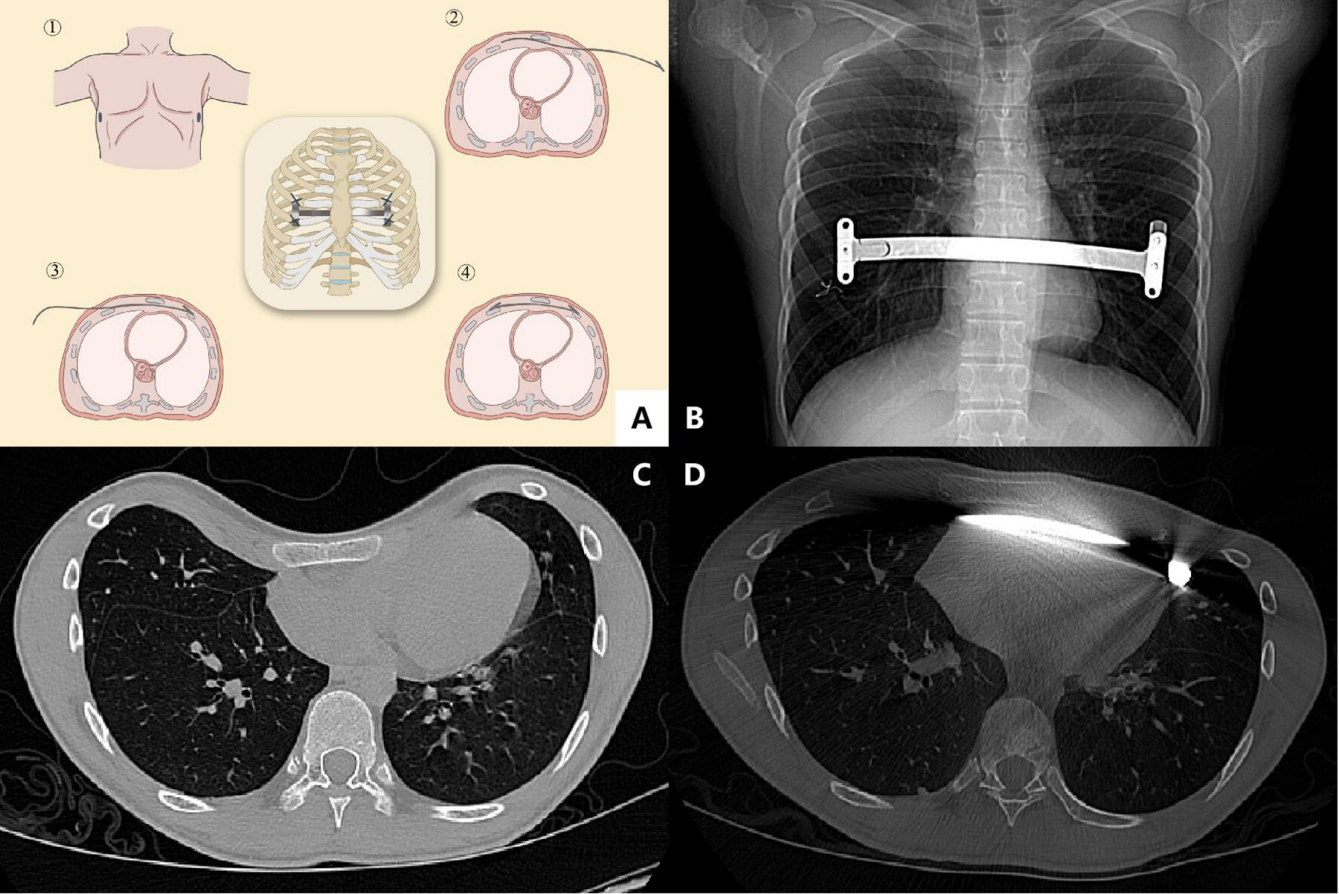
Modified Nuss procedure with non-flipping bar. **(A)** Schematic illustration of the non-flipping technique showing bilateral working incisions and advancement of a pre-shaped bar through the retrosternal tunnel in its final orientation (convex side anterior) without intraoperative 180° turnover, with bilateral stabilizer fixation (gaskets may be used when needed). **(B)** Postoperative chest radiograph demonstrating the corrective bar with bilateral stabilizers. **(C)** Preoperative axial CT image at the level of maximal sternal depression. **(D)** Postoperative axial CT confirming bar position and fixation. PE, pectus excavatum (14).

A 5-mm thoracoscope was introduced into the right pleural cavity through the sixth to eighth intercostal space at the mid-axillary line to guide and monitor the procedure. Bilateral vertical skin incisions (approximately 1.5–2.5 cm) were made near the mid-axillary line at the level corresponding to the deepest sternal depression, and subcutaneous tunnels were created toward the pectus ridge for bar passage. In patients with recurrent deformity or suspected retrosternal adhesions, a small vertical subxiphoid anterior chest wall incision was created; the surgeon's index finger was passed beneath the lower sternum to bluntly dissect retrosternal adhesions, push the pericardium away from the posterior sternal table, and provide digital protection while the passer traversed the retrosternal space. After the bar was connected to the introducer, the introducer was inserted into the right thoracic cavity and advanced behind the sternum anterior to the pericardium through the bilateral pleural cavities under thoracoscopic guidance, then delivered to the contralateral incision. The bar was pushed in and pulled through the tunnel following the introducer without turnover (“non-flipping”), thereby correcting the deformity. After the introducer was removed, stabilizer(s) were applied (with gaskets used when needed to adjust the elevation height) and secured to the ribs/intercostal muscles with wire or non-absorbable sutures to prevent rotation. Before closure, the lungs were reinflated and pleural air was evacuated using large tidal-volume ventilation and suction through the thoracoscopic trocar; chest drainage was placed selectively when extensive adhesiolysis had been performed (14, 17, 33–35) (Figure 3).

## 3 Statistical analysis

Statistical analyses were performed using R (version 4.4.0). Continuous variables were assessed for normality using the Shapiro–Wilk test and are presented as mean ± standard deviation for normally distributed data or median (interquartile range, IQR) otherwise. Categorical variables are presented as counts (percentages). Between-group comparisons were conducted using Student's t-test or the Mann–Whitney U test for continuous variables, and the chi-square test or Fisher's exact test for categorical variables, as appropriate. Age was recorded in months in the electronic medical record and converted to years (months/12) for analyses and reporting to ensure consistency. Given the broad age range of the cohort, anthropometric measures (height and weight) were considered age-dependent descriptors and were not interpreted as direct indicators of surgical outcome.

To mitigate baseline imbalances between the single-incision group and the non-flipping bar group, propensity score matching (PSM) was performed using the MatchIt package. Propensity scores were estimated using a multivariable logistic regression model including clinically relevant baseline covariates (age, sex, preoperative deformity severity and morphology metrics, and other key baseline characteristics available in the dataset). Patients were matched 1:1 using nearest-neighbor matching without replacement with a caliper of 0.2 of the standard deviation of the logit of the propensity score. Covariate balance before and after matching was evaluated using standardized mean differences (SMD), with SMD <0.10 considered acceptable balance. Primary outcome comparisons were performed in both the original cohort and the matched cohort; analyses of the matched cohort accounted for the paired nature of the data (paired tests for continuous variables and McNemar's test for categorical variables, where applicable). A two-sided p value <0.05 was considered statistically significant.

Complication definitions and severity grading. Postoperative complications were defined as any adverse event occurring after surgery and before discharge (early) or during follow-up until bar removal (late) that was attributable to the procedure, anesthesia, or the implanted devices. Findings present preoperatively (e.g., baseline cardiopulmonary anomalies) were not classified as postoperative complications. Pneumothorax and pleural effusion were recorded only when confirmed radiographically and were further categorized by whether invasive treatment was required (e.g., chest tube insertion or drainage). Surgical site complications were recorded when additional wound management (antibiotics and/or debridement) was required. Bar-related events included displacement/rotation confirmed on imaging, need for revision surgery, or premature bar removal. Complication severity was graded using the Clavien-Dindo classification (36).

## 4 Results

All 171 patients received effective surgical interventions. The single-incision group included 140 patients (103 males, 37 females) with a median age of 5.7 [4.0, 9.7] years (range: 3–16 years), a median height of 114.00 [105.00, 141.00] cm, a median weight of 20.00 [16.50, 30.12] kg, a median disease duration of 36.00 [24.00, 60.00] months, as well as median depression measurements—length: 5.00 [4.75, 6.25] cm, width: 5.00 [4.00, 6.00] cm, and depth: 2.00 [2.00, 2.50] cm. Preoperative indices were: Haller index 3.96 [3.31, 4.94], pectoralis correction index (PCI) 0.65 ± 0.12, cardiac compression index (CCI) 2.16 [1.78, 2.69], and chest wall asymmetry index (CWAI) 0.98 [0.94, 1.02]. The non-flipping bar group comprised 31 patients (30 males, 1 female) with a median age of 12.7 [10.0, 13.8] years (range: 3–17 years), median height of 161.00 [144.00, 168.50] cm, median weight of 43.50 [29.50, 49.75] kg, median disease duration of 24.00 [3.50, 60.00] months, and median depression measurements—length: 5.00 [5.00, 5.50] cm, width: 6.00 [5.00, 6.00] cm, depth: 2.00 [2.00, 3.00] cm. Preoperative indices for this group were: Haller index 4.22 [3.57, 4.78], PCI 0.65 ± 0.12, CCI 2.27 [1.85, 2.74], and CWAI 0.99 [0.95, 1.05]. Statistically significant differences were observed (*p* < 0.05) (Table 1).

**Table 1:** Baseline characteristics of enrolled patients.

Parameter	Single-incision group (*n* = 140)	Non-flipping bar group (*n* = 31)	*p*-value
Sex			
Male	103 (73.6%)	30 (96.8%)	0.010
Female	37 (26.4%)	1 (3.2%)	
Age (years)	5.7 [4.0, 9.7]	12.7 [10.0, 13.8]	<0.001
Height (cm)	114.00 [105.00, 141.00]	161.00 [144.00, 168.50]	—
Weight (kg)	20.00 [16.50, 30.12]	43.50 [29.50, 49.75]	—
Preoperative comorbidities	27 (19.3%)	4 (12.9%)	0.371
Cardiac anomalies	8 (5.7%)	1 (3.2%)	0.706
Pulmonary abnormalities	3 (2.1%)	2 (6.5%)	0.260
Upper respiratory disorders	8 (5.7%)	0 (0.0%)	0.355
Skeletal abnormalities	5 (3.6%)	0 (0.0%)	0.592
Others	3 (2.1%)	1 (3.2%)	1.000
Disease duration (months)	36.00 [24.00, 60.00]	24.00 [3.50, 60.00]	0.122
Depression length (cm)	5.00 [4.75, 6.25]	5.00 [5.00, 5.50]	0.703
Depression width (cm)	5.00 [4.00, 6.00]	6.00 [5.00, 6.00]	0.404
Depression depth (cm)	2.00 [2.00, 2.50]	2.00 [2.00, 3.00]	0.013
HI	3.96 [3.31, 4.94]	4.22 [3.57, 4.78]	0.283
PCI	0.65 ± 0.12	0.65 ± 0.12	0.799
CWAI	0.98 [0.94, 1.02]	0.99 [0.95, 1.05]	0.411
CCI	2.16 [1.78, 2.69]	2.27 [1.85, 2.74]	0.446

Height and weight are age-dependent and are presented descriptively; p values are not shown for these variables. “Cardiac anomalies” refer to pre-existing findings documented on preoperative evaluation (e.g., ECG abnormalities, mitral valve prolapse), and “pulmonary abnormalities” refer to pre-existing respiratory findings (e.g., decreased pulmonary function when available). These baseline findings were not counted as postoperative complications.

Bold values indicate statistical significance (*p* < 0.05).

Complications were categorized as early (during index hospitalization or within 30 days) and late (30 days to bar removal) events and graded using the Clavien-Dindo classification. Baseline cardiopulmonary anomalies identified during preoperative evaluation were not counted as postoperative complications. No significant differences were observed in overall complication rates between groups (*p* > 0.05). Key postoperative complications and their severity are summarized in Table 2. Notably, bar displacement/rotation requiring revision was analyzed as a postoperative bar-related complication and does not alter group assignment, which was based on the intended implantation maneuver (Table 3).

**Table 2:** Postoperative complications and Clavien–Dindo grades.

Complication	Single-incision (*n* = 140)	Non-flipping (*n* = 31)	Intervention required	Clavien-Dindo grade
Pulmonary complications (pneumothorax/pleural effusion)	25 (17.9%)	5 (16.1%)	No invasive intervention	I
Thoracic deformity requiring observation	7 (5.0%)	1 (3.2%)	Clinical follow-up only	I
Bar displacement requiring revision	4 (2.9%)	1 (3.2%)	Revision surgery with reinforcement/repositioning	IIIb
Poor wound healing requiring debridement	2 (1.4%)	0 (0.0%)	Surgical debridement	IIIb

**Table 3:** Adverse Postoperative Outcomes in Enrolled Patients.

Parameter	Single-incision group (*n* = 140)	Non-flipping bar group (*n* = 31)	*p*-value
Adverse events			
No	103 (73.6%)	25 (80.6%)	0.289
Yes	37 (26.4%)	6 (19.4%)	
Specific complications			
Thoracic deformity	7 (5.0%)	1 (3.2%)	0.693
Bar displacement	4 (2.9%)	1 (3.2%)	1.000
Pulmonary complications	25 (17.9%)	5 (16.1%)	0.804
Poor wound healing	2 (1.4%)	0 (0.0%)	1.000
Recurrence	9 (6.4%)	1 (3.2%)	0.705

Operative technique differed mainly in fixation and access. The non-flipping bar group required more than one skin incision in most cases and had a longer operative duration, whereas the single-incision group more often completed the procedure through a single working incision with shorter operative time. Screw fixation was used less frequently in the non-flipping bar group, reflecting differences in stabilization strategy (all *p* < 0.05).

Use of an additional thoracoscopic port and the number of corrective bars did not differ between groups (both 12.9% received an adjunct thoracoscopic port; nearly all patients received one bar), indicating comparable intraoperative visualization strategies and implant burden (*p* > 0.05) (Table 4).

**Table 4:** Surgical Details of Enrolled Patients.

Parameter	Single-incision group (*n* = 140)	Non-flipping bar group (*n* = 31)	*p*-value
Thoracoscopic assistance	18 (12.9%)	4 (12.9%)	0.804
Number of corrective bars			
1	138 (98.6%)	31 (100.0%)	1.000
2	2 (1.4%)	0 (0.0%)	
Number of screws			
0	74 (52.9%)	21 (67.7%)	<0.001
1	64 (45.7%)	3 (9.7%)	
≥2	2 (1.4%)	7 (22.6%)	
Number of incisions			
1	133 (95.0%)	0 (0.0%)	<0.001
2	6 (4.3%)	30 (96.8%)	
3	1 (0.7%)	1 (3.2%)	
Operative time (minutes)	55.00 [40.00, 70.00]	78.00 [58.00, 104.50]	<0.001

Postoperative morphologic indices (HI, PCI, CCI) and bar retention time were broadly comparable between groups, and overall efficacy ratings were similar. Although postoperative HI was statistically higher in the non-flipping bar group, the absolute difference was small and may have limited clinical relevance; no other postoperative endpoints differed significantly (*p* > 0.05) (Table 5).

**Table 5:** Postoperative outcomes of enrolled patients.

Parameter	Single-incision group (*n* = 140)	Non-flipping bar group (*n* = 31)	*p*-value
Postoperative HI	2.37 [2.13, 2.73]	2.65 [2.38, 3.00]	0.008
Postoperative PCI	0.93 [0.83, 0.96]	0.97 [0.94, 1.00]	0.067
Postoperative CCI	1.23 [1.08, 1.43]	1.27 [1.06, 1.34]	0.677
Bar retention time (months)	29.00 [24.00, 36.00]	30.00 [24.00, 35.00]	0.481
Surgical efficacy			
Excellent	128 (91.4%)	29 (93.5%)	0.165
Good	9 (6.4%)	0 (0.0%)	
Fair	3 (2.1%)	2 (6.5%)	

Outcome categories were defined as described in Methods: Outcome definitions.

## 5 Discussion

Our study compared two modified MIRPE strategies in 171 children and adolescents treated at a high-volume pediatric center. In the unmatched cohort, the non-flipping bar group was older with deeper deformity features, reflecting real-world selection patterns; after propensity score matching, short-to-intermediate-term efficacy and overall complication burden were comparable between approaches.

Comparison with prior literature suggests that both strategies are consistent with the broader MIRPE safety profile reported in classic and contemporary series. The original Nuss technique and subsequent large series have established high rates of cosmetic improvement and functional benefit with acceptable complication rates (8, 9). More recent work has emphasized that complications are often related to tunnel creation and bar stability, supporting the use of thoracoscopy, sternal elevation, and robust fixation as safety-focused modifications (4, 9–13).

Single-incision MIRPE has been proposed to minimize visible scarring and simplify wound care, which may be particularly attractive in younger children with more compliant chest walls (15–17). In our cohort, the single-incision strategy was associated with fewer skin incisions and shorter operative duration, without an observed increase in bar displacement requiring revision. These findings align with the concept that, in selected younger or symmetric cases, minimizing access points does not necessarily compromise safety when thoracoscopic visualization is available and mediastinal protection maneuvers (e.g., sternal elevation) are applied as needed (4, 9, 13, 15).

Limitations include the retrospective single-center design, residual confounding despite matching, imbalance in group sizes, and the relatively short follow-up that does not fully capture outcomes after bar removal and the pubertal growth spurt; therefore, true long-term recurrence rates—particularly in patients operated on at a young age—may be underestimated (7, 37–39). Future research should incorporate multicenter prospective designs, standardized patient-reported outcomes and cardiopulmonary testing, and longer follow-up through and after bar removal to clarify durability and functional benefit. In addition, detailed subgroup analyses by age, deformity morphology, and chest-wall rigidity may refine patient selection criteria for each modified technique.

Postoperative management should be interpreted as part of the technique package. Effective pain control is essential for deep breathing, early ambulation, and preventing pulmonary complications after MIRPE, and contemporary practice increasingly relies on multimodal regimens incorporating regional techniques such as intercostal nerve block in addition to patient-controlled opioid analgesia (13, 20, 40, 41). Consistent perioperative protocols in our center may have contributed to comparable early recovery between groups.

From a pragmatic standpoint, in our practice the single-incision strategy may be favored when minimizing visible scars and simplifying access is a priority and retrosternal working space is adequate (often in younger, more compliant chest walls). The non-flipping strategy may be attractive in older patients or constrained retrosternal space, where avoiding intraoperative bar turnover can simplify the critical passage step; however, this may come at the cost of additional access and fixation time. Both approaches should be viewed as safety-oriented variants within the MIRPE spectrum rather than mutually exclusive techniques.

Limitations include the retrospective single-center design, residual confounding despite matching, imbalance in group sizes, and the relatively short follow-up that does not fully capture outcomes after bar removal (7, 37–39). Future research should incorporate multicenter prospective designs, standardized patient-reported outcomes and cardiopulmonary testing, and longer follow-up through and after bar removal to clarify durability and functional benefit. In addition, detailed subgroup analyses by age, deformity morphology, and chest-wall rigidity may refine patient selection criteria for each modified technique.

Taken together, our findings suggest that both the single-incision modified Nuss procedure and the non-flipping bar technique can achieve satisfactory correction with comparable short-to-intermediate-term safety in pediatric and adolescent PE. In current practice, technique selection may reasonably be individualized based on patient age, deformity symmetry/severity, anticipated retrosternal working space, and the need for adjunct safety maneuvers.

## 6 Conclusion

The present study provides preliminary evidence that both the single-incision modified Nuss procedure and the modified Nuss procedure with non-flipping bar are effective for the management of pectus excavatum (PE) in pediatric and adolescent populations. It is encouraged to conduct more clinical studies to supplement the relevant evidence.

## Data Availability

The original contributions presented in the study are included in the article/Supplementary Material, further inquiries can be directed to the corresponding authors.

## References

[1] Notrica DM McMahon LE Jaroszewski DE. Pectus disorders: excavatum, carinatum and arcuatum. Adv Pediatr. (2024) 71(1):181–94. 10.1016/j.yapd.2024.05.001 38944483

[2] Kar A Baghai M Hunt I. Reshaping the evidence for surgical correction of pectus excavatum using cardiopulmonary exercise testing. J Am Heart Assoc. (2022) 11(7):e025273. 10.1161/JAHA.122.025273 35377161 PMC9075475

[3] Filaire L Costes F Touron J Tardy MM Molnar I Perrault H Hemodynamic changes in patients with pectus excavatum during exercise: relationship to chest wall deformity indexes. J Am Coll Cardiol. (2024) 84(15):1498–500. 10.1016/j.jacc.2024.06.039 39357944

[4] Janssen N Daemen JHT van Polen EJ Coorens NA Jansen YJL Franssen A Pectus excavatum: consensus and controversies in clinical practice. Ann Thorac Surg. (2023) 116(1):191–9. 10.1016/j.athoracsur.2023.02.059 36997016

[5] Jaroszewski DE Farina JM Gotway MB Stearns JD Peterson MA Pulivarthi V Cardiopulmonary outcomes after the Nuss procedure in pectus excavatum. J Am Heart Assoc. (2022) 11(7):e022149. 10.1161/JAHA.121.022149 35377159 PMC9075480

[6] Sonaglioni A Nicolosi GL Trevisan R Lombardo M Grasso E Gensini GF The influence of pectus excavatum on cardiac kinetics and function in otherwise healthy individuals: a systematic review. Int J Cardiol. (2023) 381:135–44. 10.1016/j.ijcard.2023.03.058 37003372

[7] Zeineddine RM Botros M Shawwaf KA Moosavi R Aly MR Farina JM Does a high haller index influence outcomes in pectus excavatum repair? J Thorac Cardiovasc Surg. (2024) 168(5):1395–402. 10.1016/j.jtcvs.2024.04.005 38608864

[8] Nuss D Kelly RE Jr. Croitoru DP Katz ME. A 10-year review of a minimally invasive technique for the correction of pectus excavatum. J Pediatr Surg. (1998) 33(4):545–52. 10.1016/S0022-3468(98)90314-1 9574749

[9] Kelly RE Jr. Obermeyer RJ Goretsky MJ Kuhn MA Frantz FW McGuire MM Recent modifications of the Nuss procedure: the pursuit of safety during the minimally invasive repair of pectus excavatum. Ann Surg. (2022) 275(2):e496–e502. 10.1097/SLA.0000000000003877 32224740

[10] Janssen N Daemen JHT Franssen A Coorens NA Hulsewé KWE Vissers YLJ Raising the bar in the management of pectus excavatum. Transl Pediatr. (2023) 12(6):1059–62. 10.21037/tp-23-236 37427063 PMC10326747

[11] Kim H Rim G Park HJ. Technical advances in pectus bar stabilization in chest wall deformity surgery: 10-year trends and an appraisal with 1,500 patients. J Chest Surg. (2023) 56(4):229–37. 10.5090/jcs.22.136 37096252 PMC10345656

[12] Shah M Frye R Marzinsky A Phillips MR Adamson W McLean SE. Complications associated with bar fixation after Nuss repair for pectus excavatum. Am Surg. (2016) 82(9):781–2. 10.1177/000313481608200934 27670561 PMC5144925

[13] Velazco CS Arsanjani R Jaroszewski DE. Nuss procedure in the adult population for correction of pectus excavatum. Semin Pediatr Surg. (2018) 27(3):161–9. 10.1053/j.sempedsurg.2018.05.002 30078487

[14] Li G Jiang Z Xiao H Wang M Hu F Xie X A novel modified Nuss procedure for pectus excavatum: a new steel bar. Ann Thorac Surg. (2015) 99(5):1788–92. 10.1016/j.athoracsur.2014.12.060 25952207

[15] Clark JJ Johnson SM. Single incision Nuss procedure for pectus excavatum. Pediatr Surg Int. (2011) 27(7):733–6. 10.1007/s00383-011-2876-6 21387106

[16] Papandria D Arlikar J Sacco Casamassima MG Ortega G Salazar JH Zhang Y Increasing age at time of pectus excavatum repair in children: emerging consensus? J Pediatr Surg. (2013) 48(1):191–6. 10.1016/j.jpedsurg.2012.10.036 23331814

[17] Coughlin AC Ahsanuddin S Inglesby D Fox C Xu H Margulies I When to Nuss? Patient age as a risk factor for complications of minimally invasive repair of pectus excavatum: a systematic review and meta-analysis. Pediatr Surg Int. (2022) 38(3):365–75. 10.1007/s00383-021-05049-z 35006367

[18] Huerta CT Alligood DM Davis JK Ramsey WA Cobler-Lichter MD Shagabayeva L Outcomes after pectus Excavatum repair: a nationwide comparison of Nuss versus ravitch operations. J Surg Res. (2024) 303:381–9. 10.1016/j.jss.2024.09.025 39418960

[19] Li H Wang F Ji G Teng J Liang X Liang X Modified Nuss procedure for the treatment of pectus excavatum: experience of 259 patients. Asian J Surg. (2023) 46(2):692–7. 10.1016/j.asjsur.2022.06.080 35803891

[20] Burton DMH Boretsky KR. A comparison of paravertebral nerve block catheters and thoracic epidural catheters for postoperative analgesia following the Nuss procedure for pectus excavatum repair. Paediatr Anaesth. (2014) 24(5):516–20. 10.1111/pan.12369 24612096

[21] Narkhojayev N Turmetov I Kemelbekov K Bektayev E Akhmetov A Zhunissov B. Results of surgical treatment of pectus excavatum in children and adolescents. Georgian Med News. (2024) (352–353):118–22. 39441281

[22] Cruz-Centeno N Fraser JA Stewart S Marlor DR Oyetunji TA St Peter SD. Determining the optimal technique for bar fixation in the repair of pectus excavatum. J Laparoendosc Adv Surg Tech A. (2024) 34(4):368–70. 10.1089/lap.2023.0233 38150213

[23] Sujka JA St Peter SD. Quantification of pectus excavatum: anatomic indices. Semin Pediatr Surg. (2018) 27(3):122–6. 10.1053/j.sempedsurg.2018.05.006 30078482

[24] Durry A Gomes Ferreira C Tricard T Gicquel P Becmeur F. Minimally invasive repair of pectus excavatum in children: results of a modified Nuss procedure. Ann Chir Plast Esthet. (2017) 62(1):8–14. 10.1016/j.anplas.2016.10.001 27823841

[25] Yu SP Lai PS Pan CT Huang PM. Comparison of several alternatives for the management of severe pectus excavatum in the Nuss procedure. Asian J Surg. (2021) 44(5):738–41. 10.1016/j.asjsur.2020.12.039 33781681

[26] van Braak H de Beer SA Al Ghouch Y Zwaveling S Oomen MWN van Heurn LWE 15 years of vacuum bell therapy for pectus excavatum: long-term outcomes and influencing factors. J Pediatr Surg. (2025) 60(2):161891. 10.1016/j.jpedsurg.2024.161891 39306540

[27] Mendonça RP Estrela C Bueno MR Carvalho T Estrela LRA Chilvarquer I. Principles of radiological protection and application of ALARA, ALADA, and ALADAIP: a critical review. Braz Oral Res. (2025) 39:e14. 10.1590/1807-3107bor-2025.vol39.014 39936713 PMC11808696

[28] Song J Wang Q Pan Z Wu C Li Y Wang G The safety and efficacy of the modified single incision non-thoracoscopic Nuss procedure for children with pectus Excavatum. Front Pediatr. (2022) 10:831617. 10.3389/fped.2022.831617 35211432 PMC8861268

[29] Ingino CA Raggio I Toselli L Farina J Bellia-Munzón G Martínez Ferro M. Specific electrocardiographic findings in patients with pectus excavatum. Rev Esp Cardiol (Engl Ed. (2023) 76(1):62–5. 10.1016/j.recesp.2022.04.018 35667564

[30] Nuss D. Recent experiences with minimally invasive pectus excavatum repair “Nuss procedure”. Jpn J Thorac Cardiovasc Surg. (2005) 53(7):338–44. 10.1007/s11748-005-0047-1 16095232

[31] Hebra A Swoveland B Egbert M Tagge EP Georgeson K Othersen HB Jr. Outcome analysis of minimally invasive repair of pectus excavatum: review of 251 cases. J Pediatr Surg. (2000) 35(2):252–7. 10.1016/S0022-3468(00)90019-8 10693675

[32] Park HJ Lee SY Lee CS Youm W Lee KR. The Nuss procedure for pectus excavatum: evolution of techniques and early results on 322 patients. Ann Thorac Surg. (2004) 77(1):289–95. 10.1016/S0003-4975(03)01330-4 14726081

[33] Liu S Wang L Zhang H Zeng W Hu F Xiao H Modified Nuss procedure with a novel steel bar in patients with pectus excavatum post-congenital heart surgery. Interact Cardiovasc Thorac Surg. (2022) 34(3):424–30. 10.1093/icvts/ivab284 34661678 PMC8860435

[34] Wang L Liu J Li Y Feng T Cao B Xiao H Modified Nuss operation using introducer-bar complex for pectus excavatum in adults: a retrospective study. J Cardiothorac Surg. (2021) 16(1):267. 10.1186/s13019-021-01624-6 34551817 PMC8456631

[35] Wang L Bi R Xie X Xiao H Hu F Jiang L. A modfied Nuss procedure for recurrent pectus excavatum of adults. Front Surg. (2021) 8:814837. 10.3389/fsurg.2021.814837 35155553 PMC8825472

[36] Golder H Casanova D Papalois V. Evaluation of the usefulness of the Clavien-Dindo classification of surgical complications. Cir Esp (Engl Ed. (2023) 101(9):637–42. 10.1016/j.ciresp.2023.01.012 36781046

[37] Ishimaru T Kitano Y Uchida H Kawashima H Gotoh C Satoh K Growth spurt-related recurrence after Nuss procedure. J Pediatr Surg. (2009) 44(8):E13–6. 10.1016/j.jpedsurg.2009.04.014 19635285

[38] Pacheco KA Thyssen JP. Contact dermatitis from biomedical devices, implants, and metals-trouble from within. J Allergy Clin Immunol Pract. (2024) 12(9):2280–95. 10.1016/j.jaip.2024.07.016 39067854

[39] Fortmann C Göen T Wiesner S Hegermann J Kiblawi R Dohna M Titanium nitride coating of pectus bar increases metal contamination after minimally-invasive repair of pectus excavatum. PLoS One. (2023) 18(10):e0292616. 10.1371/journal.pone.0292616 37824548 PMC10569521

[40] Ma B Sun Y Hao C Liu X Shen S. Patient-Controlled intravenous analgesia with or without ultrasound-guided bilateral intercostal nerve blocks in children undergoing the Nuss procedure: a randomized, double-blinded, controlled trial. Pain Res Manag. (2022) 2022:5776833. 10.1155/2022/5776833 35910406 PMC9337970

[41] Van Polen EJ Franssen CJ Daemen JHT Isabella AJ Franssen A Hulsewé KWE Postoperative pain management after minimally invasive repair of pectus excavatum: a systematic review and network meta-analysis. J Pediatr Surg. (2025) 60(6):162282. 10.1016/j.jpedsurg.2025.162282 40122203

